# Evaluation of a Novel Flexible Cage System for C5–C6 Fixation: A Finite Element Study Against Conventional ACDF Implants

**DOI:** 10.3390/bioengineering13040375

**Published:** 2026-03-24

**Authors:** Seongho Woo, Won Mo Koo, Kinam Park, Jong-Moon Hwang, Sungwook Kang

**Affiliations:** 1 Department of Physical Medicine and Rehabilitation, Daegu Fatima Hospital, Daegu-si 41121, Republic of Korea; penguin0304@naver.com (S.W.); kxxvvm@naver.com (W.M.K.); pkn6174@gmail.com (K.P.); 2 Department of Physical Medicine and Rehabilitation, Haengbokhan Rehabilitation Medicine Clinic, Daegu-si 42174, Republic of Korea; 3 Department of Smart Ocean Mobility Engineering, Changwon National University, Changwon-si 51140, Republic of Korea

**Keywords:** anterior cervical discectomy and fusion (ACDF), cervical cage with plate (CCP), zero-profile stand-alone cage (ZPSC), flexible plate cage system (FPCS)

## Abstract

Cervical spondylosis is a common cause of spinal cord dysfunction, and anterior cervical discectomy and fusion (ACDF) is widely employed when conservative treatment fails. Conventional implant systems such as the cervical cage with plate (CCP) and zero-profile stand-alone cage (ZPSC) are commonly used to enhance spinal stability and promote fusion, but they are associated with complications including dysphagia and adjacent segment degeneration. To address these limitations, a novel flexible plate cage system (FPCS) has been developed to optimize biomechanical performance while minimizing surgical risk. In this study, a finite element model of the C3–T1 cervical spine was constructed to simulate ACDF at the C5–C6 level using CCP, ZPSC, and FPCS implants. Under standardized loading conditions, von Mises stress was analyzed in the bone, intervertebral disc, endplates, cage, and screws, using the mean of the top 5% stress values to ensure accuracy. All surgical models showed increased stress compared to the intact reference spine. The ZPSC model exhibited the highest stress in the cage and screws, suggesting a more concentrated load path. The CCP model showed a more evenly distributed stress profile, particularly affecting the inferior adjacent segment. The FPCS model demonstrated moderate cage stress, reduced screw stress, and the highest plate stress, indicating a design that effectively redirects mechanical load from the screw-bone interface toward the anterior plate. This may be related to the unique structural configuration of the FPCS, which secures screws horizontally into the anterior vertebral body without penetrating the endplates. These findings suggest that the FPCS may offer a biomechanically favorable alternative to existing ACDF implants.

## 1. Introduction

Cervical spondylosis represents a major degenerative condition of the cervical spine and is a common cause of progressive neurological impairment. When nonoperative treatment fails to provide adequate symptom relief, ACDF is widely used to decompress neural elements and stabilize the affected segment [1,2,3,4]. Implant systems such as CCP and ZPSC are frequently employed to maintain alignment and promote fusion; however, their use is associated with postoperative complications, including dysphagia, soft-tissue irritation, and adjacent segment degeneration. Although postoperative dysphagia often resolves within several months, persistent symptoms have been reported in a clinically relevant proportion of patients [5,6,7,8,9,10,11,12,13,14,15].

Implant design has been recognized as an important factor influencing these complications [16,17]. Anterior implant prominence, screw trajectory, and load-sharing characteristics all contribute to stress distribution at the bone–implant interface and surrounding soft tissues. As a result, alternative fixation strategies have been explored to reduce anterior profile while preserving mechanical stability.

The FPCS was introduced as an alternative approach that integrates an anterior plate with a cage in a low-profile anterior configuration designed to minimize anterior implant protrusion toward the prevertebral soft tissues. By altering screw orientation and load transfer mechanisms, the FPCS is intended to reduce stress concentrations at critical interfaces while maintaining sufficient fixation strength and simplifying surgical handling. Despite these proposed advantages, the biomechanical behavior of the FPCS has not been fully evaluated.

Excessive von Mises stress at the bone–implant interface is closely associated with complications such as cage subsidence and screw loosening, which may compromise fusion stability and necessitate revision surgery [18]. Accordingly, the present study used finite element analysis to compare the stress distribution of CCP, ZPSC, and FPCS constructs following ACDF at the C5–C6 level. The aim was to clarify implant-specific load-sharing patterns and their potential implications for clinical performance.

Because implant-related complications after ACDF, such as cage subsidence, screw loosening, postoperative dysphagia, and altered adjacent-segment loading, are closely associated with load transfer at the bone-implant interface, implant performance in this study was interpreted primarily on the basis of stress redistribution across the cage-endplate-plate-screw construct.

## 2. Materials and Methods

### 2.1. Development of the Finite Element Model

**Figure 1:**
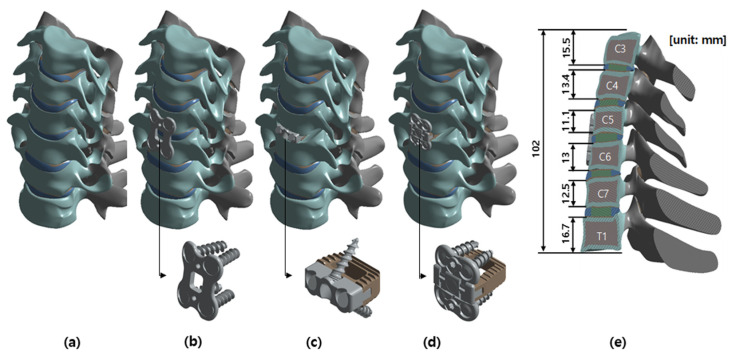
Analysis model: (**a**) Reference model, (**b**) CCP implant model, (**c**) ZPSC implant model, (**d**) FPCS implant model, (**e**) Section view of the CCP implant model.

**Figure 2:**
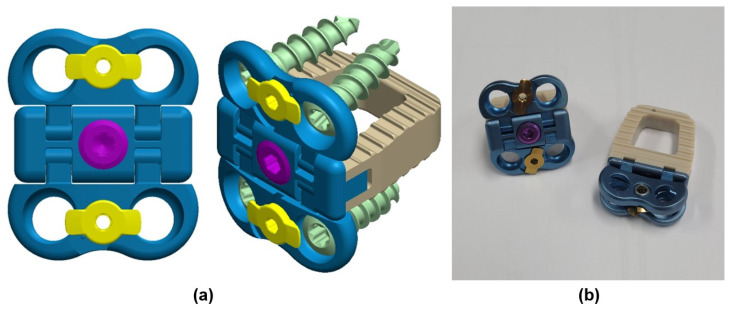
FPCS model: (**a**) 3D models of FPCS implant; (**b**) Exhibit mockup of FPCS implant.

A three-dimensional finite element method (FEM) analysis was conducted to investigate the effects on the third cervical (C3) to the first thoracic (T1) vertebrae under three conditions: surgery with a cervical cage with plate (CCP) implant, surgery with a ZPSC implant, surgery with an FPCS implant, and a reference case (without surgery) [19]. The model included the C3-T1 vertebrae, encompassing both cortical and cancellous bones, as well as the intervertebral discs, which comprised the annulus fibrosus and nucleus pulposus. Additionally, the model incorporated the endplates, including both the upper and lower endplates, and the posterior elements, such as the pedicles, laminae, and facet joints. The cage system, illustrated in Figure 1, consisted of screws, plates, and artificial discs. The spine was modeled using data from previous studies and the finite element model has already been validated in an existing paper [7,20,21]. The cage system was positioned anteriorly, midway between the fifth (C5) and sixth (C6) cervical vertebrae. Figure 2 illustrates the FPCS implant used in this study, including the three-dimensional CAD geometry and a physical mockup. The configuration highlights the integrated plate–cage design and the screw trajectory adopted for fixation at the C5–C6 level. No patients were included as subjects in the study. ANSYS SpaceClaim software 2024 R2 (SpaceClaim Corporation, Concord, MA, USA) was utilized to modify the three-dimensional (3D) models.

The intact cervical spine model used in this study was based on previously published and validated cervical finite element frameworks and served as a common host model for all postoperative constructs. To isolate implant-design effects under identical modeling assumptions, the CCP and ZPSC models were defined as representative configurations of commonly used anterior cervical constructs, whereas the FPCS model corresponded to the prototype shown in Figure 1 and Figure 2. CCP was modeled as a separate cage-plus-anterior-plate construct, ZPSC as an integrated anchored cage construct, and FPCS as a low-profile plate-cage construct with horizontal screw fixation.

### 2.2. Mesh and Material Properties for the FEM

Finite element analysis was performed using the ANSYS Workbench Static Structural module. The models were meshed with 10-node quadratic tetrahedral elements, with a characteristic element size of 0.7 mm for the implant system and 1.0 mm for the remaining spinal structures. Mesh convergence was evaluated using the nodal maximum von Mises stress at the location of the highest stress concentration; after additional mesh refinement, the difference in peak nodal stress was less than 3%, confirming convergence. The total mesh counts were 376,885 nodes and 206,809 elements for the intact reference model, 451,485 nodes and 244,247 elements for the CCP model, 415,591 nodes and 226,690 elements for the ZPSC model, and 449,238 nodes and 244,013 elements for the FPCS model. Material properties were assumed to be homogeneous, isotropic, and linearly elastic, as summarized in Table 1. The plate and screws were modeled as titanium, and the cage was modeled as PEEK.

### 2.3. Loading and Boundary Conditions

**Figure 3:**
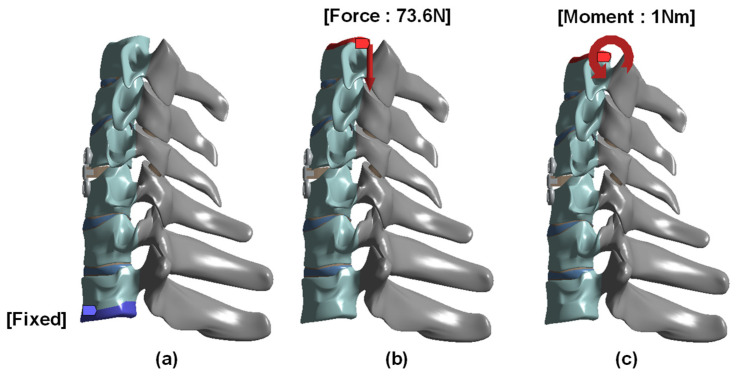
Geometry boundary and loading condition: (**a**) Fixed support at the inferior surface of T1; (**b**) Superiorly directed compressive force of 73.6 N; and (**c**) Flexion moment of 1 Nm.

A vertical compressive load of 73.6 N and a 1.0 Nm moment were applied to the superior surface of C3, while the inferior surface of T1 was fixed [21,22,23,25,26]. A physiological follower load was not applied. Except for bolt-thread contact, the implant-bone interfaces were assumed to be bonded. The analysis was limited to flexion because the objective was to compare anterior-column load transfer across the cage-endplate-plate-screw construct under a representative loading condition. The boundary and loading conditions applied in the finite element model are shown in Figure 3.

### 2.4. Outcome Measures

Representative von Mises stress was used as the primary outcome measure and was reported as the mean of the top 5% highest nodal stresses to reduce sensitivity to local numerical singularities. Endplate subsidence was not simulated directly; instead, subsidence risk was inferred from stress concentration in the endplates adjacent to the cage. Segmental range of motion (ROM) was not extracted as a separate endpoint, and stability in this study therefore refers to comparative load-sharing behavior under the applied loading condition.

## 3. Results

In this study, to ensure reliable evaluation and to avoid overestimation due to localized singularities, the von Mises stress was calculated as the mean of the top 5% highest values among all nodal stresses in the model, following the methodology established by Versluis et al. [19,24,27,28,29,30,31,32]. This volume-based approach mitigates the influence of mesh-dependent artifacts and provides a representative value for the critical material volume prone to failure initiation. The representative von Mises stress on the cortical bone, cancellous bone, upper and lower endplates, intervertebral discs, cage, and screws was evaluated as the mean of the top 5% highest nodal stress values across four models: the reference model (no surgery), the CCP model (surgery with CCP implant), the ZPSC model (surgery with ZPSC implant), and the FPCS model (surgery with FPCS implant). Table 2 provides a summary of these values, in which percentage variations for vertebral and disc components are calculated relative to the intact reference model, whereas percentage variations for implant components (cage, plate, and screws) are calculated relative to the CCP model.

**Figure 4:**
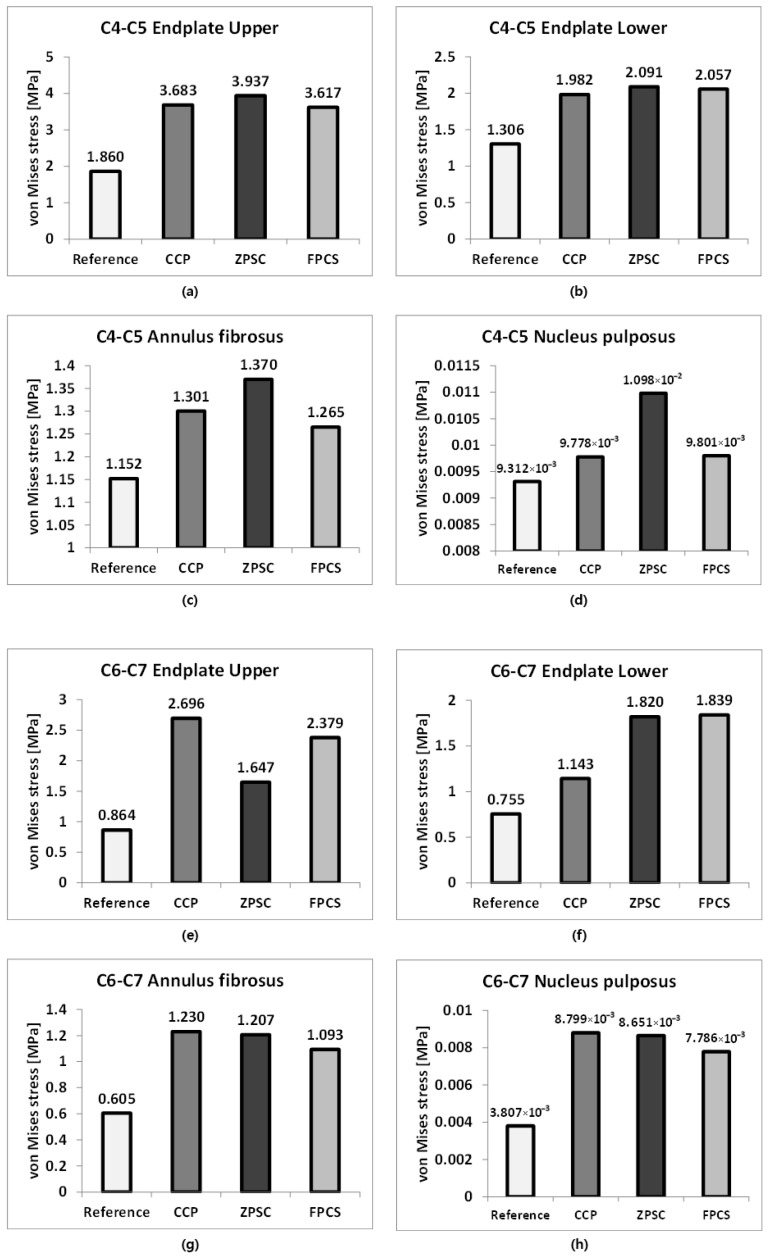

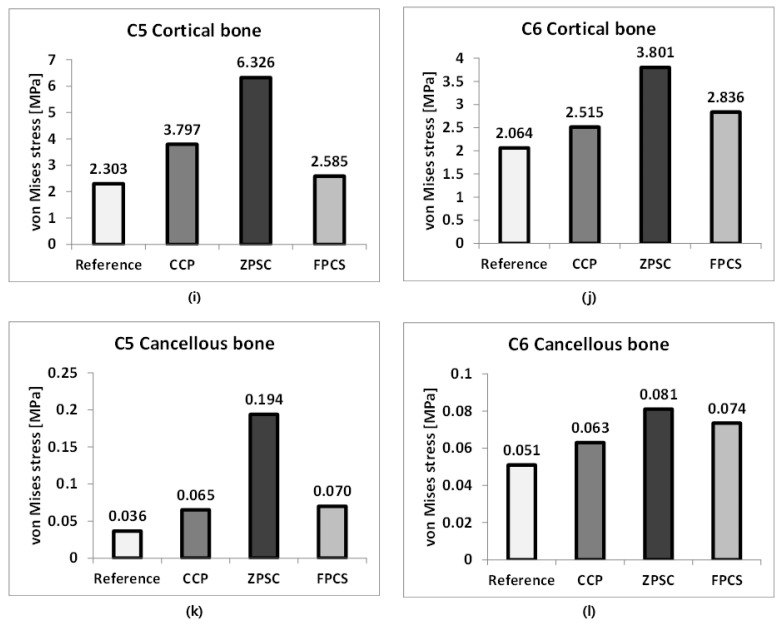
Analysis results: (**a**) C4–C5 upper endplate; (**b**) C4–C5 lower endplate; (**c**) C4–C5 annulus fibrosus; (**d**) C4–C5 nucleus pulposus; (**e**) C6–C7 upper endplate; (**f**) C6–C7 lower endplate; (**g**) C6–C7 annulus fibrosus; (**h**) C6–C7 nucleus pulposus; (**i**) C5 cortical bone; (**j**) C6 cortical bone; (**k**) C5 cancellous bone; (**l**) C6 cancellous bone.

**Figure 5:**
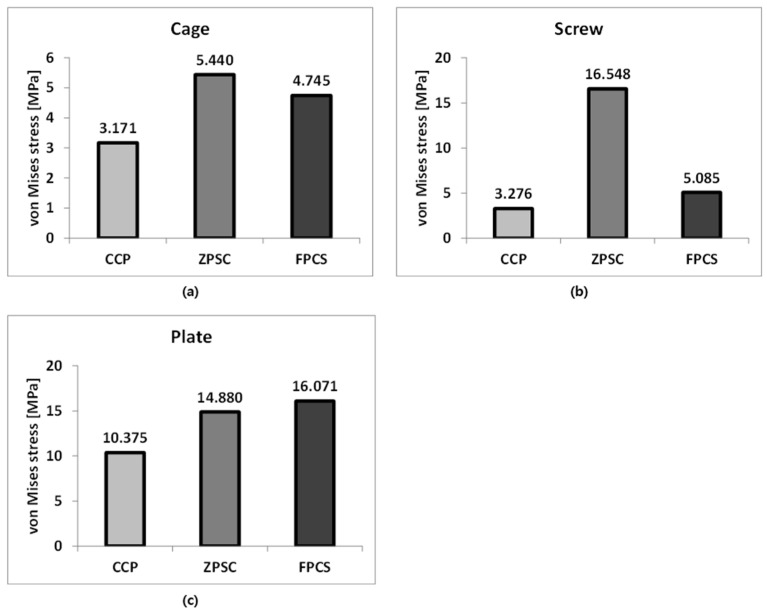
Analysis Results: (**a**) Cage; (**b**) Screw; (**c**) Plate.

**Figure 6:**
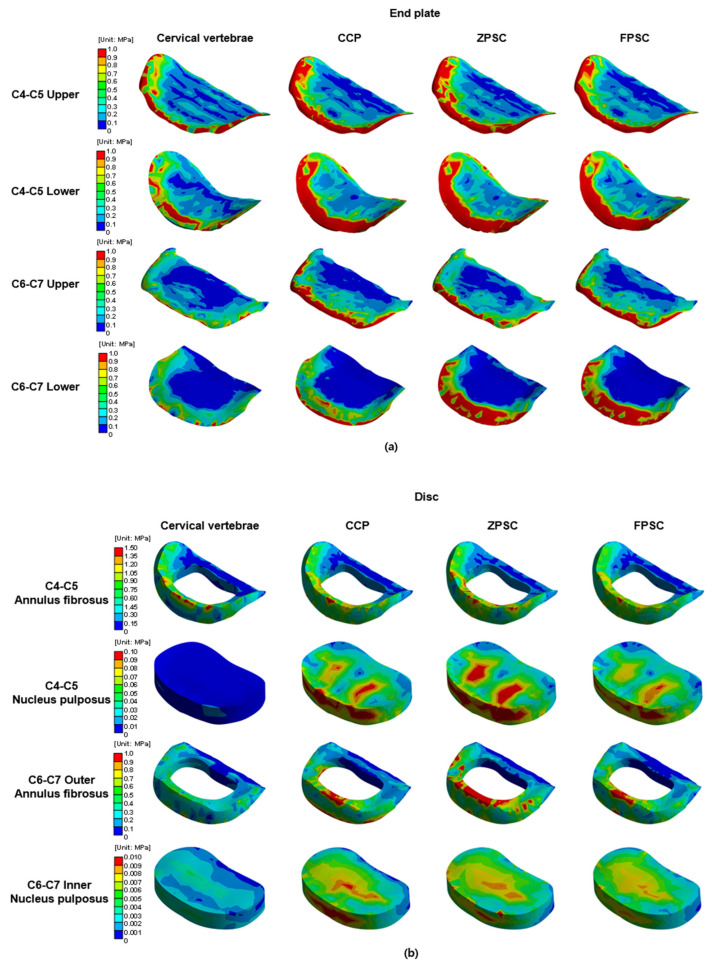

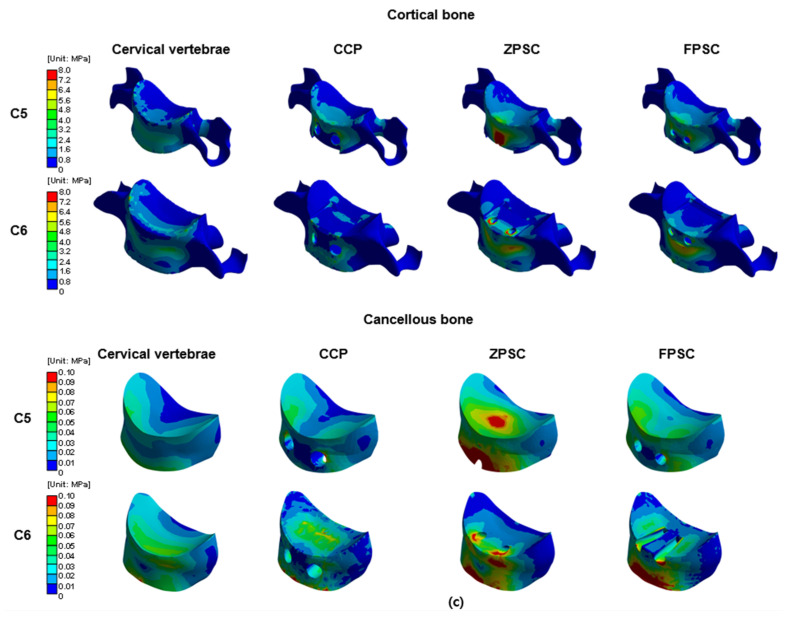
Von Mises stress results at each structure in three different models (Unit: MPa): (**a**) upper and lower endplate; (**b**) intervertebral disc; (**c**) cortical and cancellous bone.

**Figure 7:**
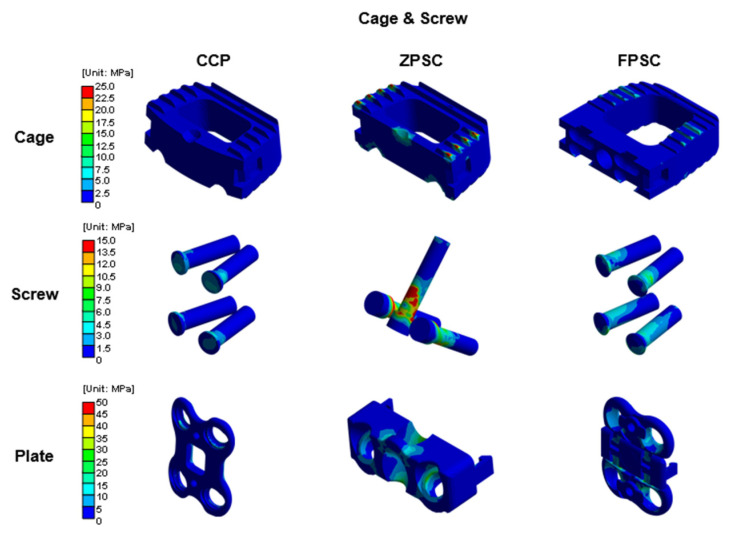
Von Mises stress results at cage and screw in three different models (Unit: MPa).

Figure 4, Figure 5, Figure 6 and Figure 7 provide a visual summary of the stress distribution results across the models (spinal structures and implant components) for ease of comparison.

### 3.1. Cortical and Cancellous Bone Stress Analysis at the ACDF on C5–C6 Segment

The representative von Mises stress values (top 5% average) in the cortical and cancellous bones at the C5 and C6 levels were analyzed across all models.

At C5 cortical bone, the von Mises stress increased from 2.303 MPa in the reference model to 3.797 MPa (164.87%) in the CCP model and 6.326 MPa (274.69%) in the ZPSC model. In contrast, the FPCS model showed a lower stress value of 2.585 MPa (112.23%) compared to the reference.

At C5 cortical bone, the von Mises stress increased from 2.303 MPa in the reference model to 3.797 MPa (164.87%) in the CCP model and 6.326 MPa (274.69%) in the ZPSC model. In contrast, the FPCS model showed a lower stress value of 2.585 MPa (112.23%) compared to the reference. For C6 cortical bone, stress increased from 2.064 MPa in the reference model to 2.515 MPa (121.85%) in the CCP model, 3.801 MPa (184.16%) in the ZPSC model, and 2.836 MPa (137.40%) in the FPCS model.

In the case of C5 cancellous bone, the von Mises stress rose from 0.036 MPa in the reference model to 0.065 MPa (178.68%) in the CCP model and 0.194 MPa (531.65%) in the ZPSC model. The FPCS model showed an intermediate increase to 0.070 MPa (192.00%). At C6 cancellous bone, the stress increased from 0.051 MPa in the reference model to 0.063 MPa (123.53%) in the CCP model, 0.081 MPa (158.82%) in the ZPSC model, and 0.074 MPa (144.12%) in the FPCS model. All surgical models exhibited higher stresses than the reference.

### 3.2. Stress Distribution in Adjacent Segments (C4–C5 and C6–C7) Following ACDF at C5–C6

The representative von Mises stress values for the C4–C5 intervertebral disc were evaluated across four regions: the upper endplate, lower endplate, annulus fibrosus, and nucleus pulposus. In the reference model, stress values were 1.860, 1.306, 1.152, and 9.312 × 10^−3^ MPa, respectively. The CCP model showed increased upper and lower endplate stress to 3.683 and 1.982 MPa, and elevated stresses in the annulus fibrosus and nucleus pulposus to 1.301 and 9.778 × 10^−3^ MPa. The ZPSC model further increased the upper and lower endplate stresses to 3.937 and 2.091 MPa, with the annulus fibrosus and nucleus pulposus stresses measured at 1.370 and 1.098 × 10^−2^ MPa. The FPCS model exhibited slightly lower upper and lower endplate stresses of 3.617 and 2.057 MPa, and the annulus fibrosus and nucleus pulposus stresses were 1.265 and 9.801 × 10^−3^ MPa, respectively. At both adjacent segments (C4–C5 and C6–C7), all surgical models exhibited increased stress compared to the reference. Notably, distinct stress patterns were observed among the implants: the ZPSC model consistently induced the highest stress concentrations at the superior segment (C4–C5), whereas the CCP model predominantly resulted in the highest stress at the inferior segment (C6–C7). In contrast, the FPCS model demonstrated an intermediate stress profile, maintaining relatively moderate values across most regions, albeit with a slight elevation at the C6–C7 lower endplate as noted in the Discussion.

These findings suggest that all implant models increase stress in adjacent segments compared to the reference, with ZPSC inducing the highest stress concentrations at the superior segment (C4–C5), while CCP predominantly affects the inferior segment (C6–C7). The FPCS model, although increasing stress overall, tended to produce relatively moderate stress values across most regions.

### 3.3. Implant Stress Characteristics of CCP, ZPSC, and FPCS at the C5–C6 ACDF

The representative von Mises stress values on the implants were analyzed to assess mechanical loading characteristics and potential stress concentration among the three surgical models. For the cage component, the ZPSC model exhibited the highest stress at 5.440 MPa, followed by the FPCS model at 4.745 MPa. The CCP model showed the lowest cage stress at 3.171 MPa, indicating that in this design, less load is transmitted through the cage.

Regarding the screw component, the ZPSC model showed the highest stress at 16.548 MPa, followed by the FPCS model at 5.085 MPa, and the CCP model at 3.276 MPa. These results suggest that the ZPSC model may experience greater mechanical demand at the screw interface, which could influence long-term fatigue performance.

For the plate component, the highest stress was observed in the FPCS model at 16.071 MPa, followed by ZPSC at 14.880 MPa, and CCP at 10.375 MPa. This indicates that the FPCS design transfers more load through the anterior plate, potentially providing better stability while reducing stress on the screw.

## 4. Discussion

This study aimed to evaluate the biomechanical performance of a novel FPCS in ACDF at the C5–C6 level, using FEA to compare it with two widely used systems: the CCP and the ZPSC [7,18,19]. Von Mises stress distribution was analyzed in the vertebrae, intervertebral discs, endplates, and implant components to assess mechanical load transfer and potential clinical implications.

All surgical models resulted in elevated stress levels in both the vertebral structures and adjacent segments compared to the intact reference model, consistent with the biomechanical consequences of fusion procedures. The ZPSC model showed the highest stress concentrations overall, particularly in the C5 vertebra, screw, and cage [11,15,33,34,35,36,37]. This pattern suggests a concentrated load path and potentially increased risk for implant-related complications such as screw loosening or cage subsidence. The rigid, compact design of ZPSC, which anchors screws obliquely through the vertebral endplate, may contribute to these high localized stresses, especially in adjacent levels where mobility is preserved.

The CCP model demonstrated a more evenly distributed stress profile, with relatively lower stress in implant components. However, it induced notable stress increases at the C6–C7 level, raising concerns regarding inferior adjacent segment degeneration [14,38]. While CCP systems benefit from strong fixation and well-established clinical outcomes, their prominent anterior profile has been associated with soft tissue irritation and postoperative dysphagia [11,12,38,39].

The FPCS model also exhibited lower stress at C5–C6 bone stress, and a favorable balance in stress distribution. Notably, it showed moderate stress within the cage, low screw stress, and the highest stress in the anterior plate among all models. These findings imply a biomechanical strategy in which the FPCS design intentionally redirects load-bearing from the screw-bone interface toward the anterior plate. This may reduce the risk of screw fatigue and improve long-term implant stability. Importantly, the structural characteristics of FPCS likely contribute to this performance. Unlike ZPSC, which fixes screws through the endplate, the FPCS anchors screws horizontally into the anterior portion of the vertebral body, avoiding direct penetration of the endplate and potentially reducing stress concentrations [11,33,40,41,42,43]. Additionally, by integrating an anterior plate and an interbody cage within a stream-lined implant configuration, the FPCS seeks to balance the mechanical stability associated with CCP while reducing anterior hardware prominence.

In summary, the ZPSC model exhibited the highest implant stresses overall, particularly in the screw and cage, suggesting a more concentrated load path. These findings are consistent with previous biomechanical studies; for instance, Ahn et al. (2023) [7] reported significantly higher screw and cage stresses in ZPSC constructs compared to plate systems, while Ni et al. (2025) [43] highlighted the susceptibility of zero-profile screws to loosening. Clinically, this stress concentration mirrors the higher subsidence rates reported in stand-alone cages by Han et al. (2016) [44] and Jin et al. (2021) [15]. Conversely, the CCP model showed the most evenly distributed stress with relatively low values across all components, a pattern supported by Lin et al. (2021) [45], who observed that anterior plating effectively reduces localized stress and micromotion. Building on these validated baselines, the FPCS model demonstrated moderate cage stress, low screw stress, and the highest plate stress. This indicates a design optimized to shift mechanical loading toward the anterior plate while minimizing stress on the screws. The structural orientation and screw trajectory of the FPCS likely play a key role in achieving this biomechanical advantage. However, it is noteworthy that the FPCS exhibited the highest stress at the C6–C7 lower endplate (1.839 MPa). This specific increase suggests a localized load shift where the rigid anterior support transmits a slightly higher load to the inferior adjacent segment compared to other models, representing a trade-off for the enhanced stability at the fusion site.

Because the present comparison focused on load transfer at the cage-endplate-screw-plate interfaces, flexion was selected as a representative condition for evaluating anterior load sharing, consistent with previous ACDF finite element studies [7,43,45]. Under this condition, the FPCS showed lower screw stress and lower fusion-level cortical bone stress than ZPSC while maintaining moderate cage stress. Extension, lateral bending, and axial rotation remain important and should be addressed in future studies.

Several limitations should be considered. This was a computational comparison based on an established cervical finite element framework and did not include new cadaveric or in vitro validation of the FPCS prototype. The loading and contact conditions were simplified: no physiological follower load was applied, loading was limited to flexion, and most implant-bone interfaces were modeled as bonded. Material properties were assumed to be homogeneous, isotropic, and linearly elastic. In addition, subsidence was inferred from endplate stress concentration rather than simulated directly, and segmental ROM, facet force, and multiaxial postoperative kinematics were not evaluated.

## 5. Conclusions

This finite element analysis demonstrates that the FPCS produces a distinct pattern of load sharing compared with conventional CCP and ZPSC constructs in single-level ACDF at C5–C6. The FPCS showed reduced stress at the screw–bone interface and a redistribution of mechanical load toward the anterior plate, while maintaining moderate stress within the cage. Although all fusion constructs increased stress in adjacent segments relative to the intact spine, the FPCS generally exhibited intermediate stress levels compared with the other systems.

Further experimental and clinical studies are needed to determine whether these biomechanical differences are associated with subsidence, screw loosening, and long-term clinical outcomes.

## Figures and Tables

**Table 1:** Mesh and material properties.

Component		Number of Nodes	Number of Elements	Elastic Modulus (MPa)	Poisson Ratio	Reference
Cortical bone		153,123	84,916	12,000	0.3	[22]
Cancellous bone		42,536	23,743	100	0.2	[22]
Posterior element		118,941	68,743	3500	0.25	[22]
Endplate		30,419	14,057	1000	0.3	[20]
Nucleus pulposus		10,396	5356	1	0.499	[20]
Annulus fibrosus		18,184	8797	4.2	0.45	[20]
Facet joint		3286	1197	11	0.4	[22]
CCP	Cage	18,471	10,310	3600 (PEEK)	0.39	[23]
	Plate	36,496	21,925	113,800 (Titanium)	0.342	[24]
	Screw	19,633	5203	113,800 (Ti)	0.342	[24]
ZPSC	Cage	19,994	11,838	3600 (PEEK)	0.39	[23]
	Plate	10,574	6133	113,800 (Ti)	0.342	[24]
	Screw	8138	1910	113,800 (Ti)	0.342	[24]
FPCS	Cage	28,833	16,912	3600 (PEEK)	0.39	[23]
	Plate	28,735	15,392	113,800 (Ti)	0.342	[24]
	Screw	14,785	4900	113,800 (Ti)	0.342	[24]

**Table 2:** von Mises stress results at each structure in four different models.

Component	von Mises Stress (MPa) (% of von Mises Stress with Reference)			
	Reference	CCP	ZPSC	FPCS
C4–C5 Endplate upper	1.860	3.683 (198.01%)	3.937 (211.67%)	3.617 (194.46%)
C4–C5 Endplate lower	1.306	1.982 (151.76%)	2.091 (160.11%)	2.057 (157.53%)
C4–C5 Annulus fibrosus	1.152	1.301 (112.89%)	1.370 (118.92%)	1.265 (109.81%)
C4–C5 Nucleus pulposus	9.321 × 10^−3^	9.778 × 10^−3^ (105.00%)	1.098 × 10^−2^ (117.91%)	9.801 × 10^−3^ (105.25%)
C6–C7 Endplate upper	0.864	2.696 (311.98%)	1.647 (190.63%)	2.379 (275.35%)
C6–C7 Endplate lower	0.755	1.143 (151.36%)	1.820 (241.06%)	1.839 (243.58%)
C6–C7 Annulus fibrosus	0.605	1.230 (203.34%)	1.207 (199.50%)	1.093 (180.71%)
C6–C7 Nucleus pulposus	3.807 × 10^−3^	8.799 × 10^−3^ (231.13%)	8.651 × 10^−3^ (227.24%)	7.786 × 10^−3^ (204.52%)
C5 Cortical bone	2.303	3.797 (164.87%)	6.326 (274.69%)	2.585 (112.23%)
C6 Cortical bone	2.064	2.515 (121.85%)	3.801 (184.16%)	2.836 (137.40%)
C5 Cancellous bone	0.036	0.065 (178.68%)	0.194 (531.65%)	0.070 (192.00%)
C6 Cancellous bone	0.051	0.063 (123.53%)	0.081 (158.82%)	0.074 (144.12%)
Cage		3.171	5.440 (71.6%)	4.745 (49.6%)
Plate		10.375	14.880 (43.4%)	16.071 (54.9%)
Screw		3.276	16.548 (405.1%)	5.085 (55.2%)

## Data Availability

The data presented in this study are available on request from the corresponding author due to privacy.
